# Exploring factors influencing the retention of nurses in a religious hospital in Taiwan: a cross-sectional quantitative study

**DOI:** 10.1186/s12912-021-00558-7

**Published:** 2021-03-12

**Authors:** Li-Hua Chiao, Chiu-Feng Wu, I-Shiang Tzeng, An-Na Teng, Ru-Wen Liao, Li Ying Yu, Chin Min Huang, Wei-Han Pan, Chu-Yueh Chen, Tsai-Tsu Su

**Affiliations:** 1 Superintendent Office, Taipei Tzu Chi Hospital, Buddhist Tzu Chi Medical Foundation, New Taipei City, Taiwan; 2 Department of Nursing, Taipei Tzu Chi Hospital, Buddhist Tzu Chi Medical Foundation, New Taipei City, Taiwan; 3 Department of Research, Taipei Tzu Chi Hospital, Buddhist Tzu Chi Medical Foundation, New Taipei City, Taiwan; 4 Department of Planning and Management, Taipei Tzu Chi Hospital, Buddhist Tzu Chi Medical Foundation, New Taipei City, Taiwan; 5 Division of Public Communication, Taipei Tzu Chi Hospital, Buddhist Tzu Chi Medical Foundation, New Taipei City, Taiwan; 6 Graduate Institute of Public Affairs, College of Social Sciences, National Taiwan University, No. 1, Sec. 4, Roosevelt Road, Taipei, 10617 Taiwan (Republic of China)

**Keywords:** Nursing staff, Intention to stay, Retention measures, Faith-based hospital, Medical humanities education, Maslow’s hierarchy of needs

## Abstract

**Background:**

Long-term deficits in the nursing labor force and high turnover rates are common in the Taiwanese medical industry. Little research has investigated the psychological factors associated with the retention of nursing staff. However, in practice, religious hospitals often provide nursing staff with education in medicine or the medical humanities to enhance their psychological satisfaction. The objective of this study was to explore factors influencing nursing staff retention in their work in relation to different levels of needs. A further objective was to investigate whether medical humanities education was associated with the retention of nursing staff.

**Methods:**

This study used self-administrated questionnaires to survey nurses working in northern areas of Taiwan. The questionnaire design was based on the six levels of Maslow’s hierarchy of needs. Participation was voluntary, and the participants signed informed consent documents. Self-administrated questionnaires were distributed to a total of 759 participants, and 729 questionnaires were returned (response rate 96.04%). Logistic regression analysis was used to estimate the impact of seniority on nurses’ reported intention to stay after adjustment for nurse characteristics (gender and age).

**Results:**

In the Pearson correlation analysis, nurses’ willingness to stay was moderately correlated with “physical needs”, “safety needs”, “love and belonging needs”, and “esteem needs” (r = 0.559, *P < 0.001*; r = 0.533, *P < 0.001*; r = 0.393, *P < 0.001*; and r = 0.476, P < 0.001, respectively). Furthermore, nurses’ willingness to stay was highly correlated with “self-actualization needs”, “beyond self-actualization needs” and “medical humanities education-relevant needs” (r = 0.707, *P < 0.001*; r = 0.728, *P < 0.001*; and r = 0.678, *P < 0.001,* respectively). We found that the odds ratios (ORs) of retention of nursing staff with less than 1 year (OR = 4.511, *P = 0.002*) or 1–3 years (OR = 3.248, *P = 0.003*) of work experience were significantly higher than that of those with 5–10 years of work experience.

**Conclusions:**

With regard to medical humanities education, we recommend adjusting training, as the compulsory activities included in the official programs are inadequate, and adjusting the number of required hours of medical humanities education. Tailoring different educational programs to different groups (especially nurses who have worked 3–5 years or 5–10 years in the case study hospital) might improve acceptance by nursing staff.

**Supplementary Information:**

The online version contains supplementary material available at 10.1186/s12912-021-00558-7.

## Background

Worldwide, there is a deficit of nursing employees [1]. As highly trained and experienced registered practical staff are key personnel in any health care establishment, a sufficient number of these staff is critical to providing patient-centered health services [2, 3]. Previous research has shown a serious deficit of health care employees, especially doctors and nurses, in many countries worldwide [4–6]. In short, the field of nursing is facing a growing deficit of employees in countries around the world, and Taiwan is no exception [7, 8]. Most medical interventions require the services of doctors, nurses, and other health professionals. Research shows that a deficit of health care employees may affect health outcomes such as quality of care, morbidity, and mortality [3–6, 9, 10]. The percentage of practicing nursing staff among all people with a nursing certificate in Taiwan is approximately 59.29% [11]. Compared to that of Canada (93.6%) and that of the USA (83.2%), the percentage of registered practical nurses in Taiwan is obviously low [11]. In addition, nurse turnover rates in Taiwan for 2009–2013 were 16.95, 17.12, 18.54, 19.03 and 16.45% [12].

Hospitals in Taiwan can be divided into three categories: medical centers, regional hospitals, and district hospitals. The locations of these types of hospitals in the environment and their building structures, number of beds, facilities, and organizational structures may be similar. However, due to differences in ownership, the management systems and environmental climates of these hospitals may vary. Hospital management systems are likely to differ across different types of hospitals. Management systems affect employees and need to be considered in terms of their job performance and the quality of work life they provide.

Previous research has shown that nursing often faces time-consuming and stressful situations [13]. Factors such as low job control, high job requirements, and deficits in supportive work relationships are associated with stress [14] and high employee turnover in nurses [15]. Research has shown that nurses report their basic needs as being related to employee satisfaction [16], overall health [17], and intention to stay. It is necessary to understand the relationship among basic needs, the quality of work life in health professions, and the intention to remain in these professions. In the study hospital, there is a humanistic culture based on the principle of seeking the ideals of truth, beauty, and goodness. Education in the humanities disciplines provides tangible benefits to the realm of nurses’ work [18]. The objective of this study was to explore factors influencing nursing staff retention in their work in relation to different levels of needs. Furthermore, this study aimed to investigate whether medical humanities education was associated with the retention of nursing staff.

## Methods

### Research design and setting

This was a cross-sectional quantitative study. The study was carried out in Taipei Tzu Chi Hospital. Taipei Tzu Chi Hospital was established in the northern part of Taiwan in 2005, with 1013 beds and a mission to safeguard life and to provide comprehensive medical treatment for all, particularly those with social determinants leading to more challenging access to health services.

### Recruitment

A total of 759 questionnaires (refer to the additional file) were sent to the clinical nursing staff of the Taipei Tzu Chi Hospital. The period of data collection was between May 4, 2017, and April 30, 2018.

### Data collection

#### Variables

The data were collected using a self-report questionnaire consisting of two sections: demographic information and employee satisfaction. The demographic data obtained included gender, age, education level, religion, seniority among nursing staff, seniority among hospital staff, and duration of any medical or human-related education and training received in the past 6 months. Anonymous questionnaires were distributed to clinical nursing staff in the general wards, intensive care wards, and oncology wards but not to staff in outpatient, emergency, and other ward services to ensure work content consistency.

#### Measures

The questionnaire content was discussed with six nursing experts, and the interviewees’ (participants’) understanding of the vocabulary was measured. The questionnaire content was created based on consensus, and the participants were asked to respond on a five-point Likert scale (ranging from 1 to 5). Three nursing experts conducted expert validity tests according to predefined inspection items such as those assessing whether the items had “clear meaning,” were “related to the investigation content,” and were “easy to answer.” The content validity index (CVI) values of each item were greater than 0.8. A few items had CVI values less than 0.8 for “easy to answer” or “exactly clear”, but the overall impact of these low values was small, indicating that the questionnaire had good content validity.

The theoretical framework for the design of the questionnaire content was Maslow’s hierarchy of needs. The participants completed the questionnaire, which assessed the following dimensions of Maslow’s hierarchy of needs: (1) Physical needs; (2) Safety needs; (3) Love and belonging needs; (4) Esteem needs; (5) Self-actualization needs; (6) Beyond self-actualization needs; (7) Medical humanities education-relevant needs; and (8) Retention-relevant needs. The participants were asked to rate their degree of satisfaction of these needs from their jobs on a scale including the options Strongly Agree (SA), Agree (A), Neutral (N), Disagree (D), and Strongly Disagree (SD), which were coded as 5, 4, 3, 2, and 1, respectively. We also conducted a pilot study with 30 employed nurses to investigate the reliability of the questionnaire. The Cronbach’s alpha (α) coefficient of the overall questionnaire was .94. The Cronbach’s α coefficients of each dimension were as follows: (1) Physical needs, .75; (2) Safety needs, .61; (3) Love and belonging needs, .83; (4) Esteem needs, .79; (5) Self-actualization needs, .83; (6) Beyond self-actualization needs, .8; (7) Medical and humanities education-relevant needs, .94; and (8) Retention-relevant needs, .9. According to the results of the investigation, the scale used in this study had good reliability.

In the present study, the employee satisfaction scale included items rated on a five-point Likert scale. It included 10 items related to the following aspects of employee satisfaction: salary, total weekly working hours, safety in the working environment, mutual assistance, acceptability of constructive advice, and learning resources and opportunities for growth. The scale design was discussed with six nursing experts, and the CVI of each related item was above .8. The participants were asked to rate their degree of satisfaction with their jobs on a scale including the options Strongly Agree (SA), Agree (A), Neutral (N), Disagree (D), and Strongly Disagree (SD), which were coded as 5, 4, 3, 2, and 1, respectively. The 10-item employee satisfaction scale showed an acceptable level of reliability (Cronbach’s α = .889).

## Statistical analysis

In this study, the relationships between the different dimensions of Maslow’s hierarchy of needs were determined by using Pearson’s correlation test, and the correlation between each dimension and intention to stay was discussed. We also performed simple and multiple regression analyses to investigate the impact of the dimensions of Maslow’s hierarchy of needs on the retention of nursing staff in a faith-based hospital in Taiwan.

Pearson correlations were used to assess relationships between all variables in the model. Chi-square tests were used to detect the differences among the different groups for the categorical variables. Linear regression analysis was used to examine the pattern of the relationships among all variables. Logistic regression analyses were used to investigate the association between the nurses’ characteristics and intention to stay in the hospital. To assess model fit, we examined the R-square and the *p* value of the F statistic (< 0.05) as fit indices. SPSS version 24.0 was used to perform descriptive statistical analysis.

### Ethical approval

This study was performed in Taipei Tzu Chi Hospital from May 2017 to April 2018 and was coordinated by the superintendent offices of Taipei Tzu Chi Hospital and Buddhist Tzu Chi Medical Foundation. This study was approved by the Institutional Review Board of Taipei Tzu Chi Hospital (IRB number: 06-X10–026).

## Results

A total of 729 nurses working in different facilities responded to the questionnaire (96.04% response rate). All analyses were performed as two-sided tests with a 0.05 significance level to explore factors influencing nursing staff retention in their work in relation to different levels of needs.

### Cross-analysis of the participants’ general characteristics and intention to stay

The overall analysis demonstrated that only 87.8% of nurses were willing or very willing to keep working (refer to Table 1). Furthermore, the majority of the participants in this analysis were females (95.6%). As shown in Table 1, the majority of the participants were under the age of 29 years (57.9%), and this age group had the second lowest intention to remain a nurse (86.7%). The percentage of participants with a graduate school education was 2.7%; this group was the least likely to report intention to stay (84.2%). Among these participants, 27.2% of the nurses had more than 10 years of work experience related to nursing. Apart from those nurses with less than 1 year of work experience, this group was the most willing to continue working (91.3%). A total of 12.6% of the participants had not received medical or human-related education or training during the past 6 months; this group was reported to have the lowest intention to remain (84.4%).

**Table 1:** General characteristics of the study participants and difference of intention to stay across different categories of participants’ characteristics

	n	percentage	Intention to stay (binary)		*P*-value
			n	percentage	
Retention					
Stay	636	87.8			
Leave	88	12.2			
Sex					
Male	31	4.4	29	93.5	0.307
Female	681	95.6	595	87.4	
Age					
Below 29	414	57.9	359	86.7	0.099
30 ~ 39	221	30.9	191	86.4	
40 ~ 49	65	9.1	63	96.9	
Over 50	15	2.1	14	93.3	
Education level					
Junior high school	4	0.6	4	100	0.327
Junior college	295	41.2	258	87.5	
Two-year college	44	6.1	43	97.7	
Two-year technical college	163	22.8	139	85.3	
University	191	26.7	168	88.0	
Graduated school	19	2.7	16	84.2	
Religion					
None	375	52.5	328	87.5	0.408
Buddhism	201	28.2	178	88.6	
Taoism	94	13.2	83	88.3	
Christianity	21	2.9	20	95.2	
Catholic	9	1.3	6	66.7	
Other	14	2.0	12	85.7	
Seniority of nursing work					
Within1 year	109	15.2	101	92.7	0.003**
1 ~ 3 year	169	23.6	152	89.9	
3 ~ 5 year	118	16.5	98	83.1	
5 ~ 10 year	125	17.5	99	79.2	
Over 10 year	195	27.2	178	91.3	
Seniority of hospital work					
Within1 year	135	18.9	124	91.9	0.067
1 ~ 3 year	194	27.1	172	88.7	
3 ~ 5 year	133	18.6	110	82.7	
5 ~ 10 year	141	19.7	119	84.4	
Over 10 year	112	15.7	103	92.0	
Time of received medical and human-related education and training in the past six months					
None	90	12.6	76	84.4	0.426
1 ~ 5 h	499	69.7	437	87.6	
6 ~ 10 h	102	14.2	94	92.2	
Over 10 h	25	3.5	22	88.0	

Note: intention to stay as response scores ≥3

Note: *p < 0.05, **p < 0.01, ***p < 0.001

### Bivariate analysis

The correlations between all the levels of Maslow’s hierarchy of needs and intention to stay in this study are displayed in Table 2. The analysis found a positive correlation between each level and intention to stay. Among them, “physiological needs”, “safety requirements”, “love and subordination needs” and a moderate intention to stay were moderately positively correlated (correlations between 0.30 and 0.50). Furthermore, “self-realization needs”, “beyond self-actualization needs” and “medical humanity education-relevant needs” were strongly positively correlated (correlations greater than 0.60), and all correlations reached statistical significance (*p* < 0.001). The results of the study showed that each level of need and medical humanities education were positively related to nurses’ intention to stay at their current jobs. This finding was consistent with the research hypothesis. The results also showed that the fulfillment of the needs on Maslow’s hierarchy and increased medical humanities education may improve the retention of nursing employees. Retention measures should consider the needs of each dimension of the hierarchy to improve the staff turnover rate.

**Table 2:** Correlations for all the levels of needs and intention to stay score

Levels of Maslow’s hierarchy of needs	Intention to stay score
Physical needs	0.559***
Safety needs	0.533***
Love and belonging needs	0.393***
Esteem needs	0.476***
Self-actualization needs	0.707***
Behind self-actualization needs	0.728***
Medical humanities education relevant	0.678***

Note: *p < 0.05, **p < 0.01, ***p < 0.001

### Multivariate regression

We constructed a multivariate regression model after controlling for adjusted age, sex, and education level. We found that the dimensions in Maslow’s hierarchy of needs had significant positive effects on intention to stay. These results are displayed in Table 3 (also adjusted for the covariates). Furthermore, we found that self-actualization needs, as a risk factor, had the most significant association with intention to stay after adjustment for covariates.

**Table 3:** The relationship between Maslow’s hierarchy of needs and intention to stay score

Variables	Intention to stay score		Intention to stay (adjusted)	
	Estimate (SE)	*P*-value	Estimate (SE)	*P*-value
Physical needs	.539(.030)	< 0.001***	.543(.030)	< 0.001***
Safety needs	.645(.038)	< 0.001***	.660(.038)	< 0.001***
Love and belonging needs	.530(.046)	< 0.001***	.556(.046)	< 0.001***
Esteem needs	.590(.041)	< 0.001***	.598(.041)	< 0.001***
Self-actualization needs	.798(.030)	< 0.001***	.800(.030)	< 0.001***
Behind self-actualization needs	.715(.025)	< 0.001***	.719(.026)	< 0.001***
Medical humanities education relevant	.603(.024)	< 0.001***	.599(.025)	< 0.001***

Adjusted age, sex, education level in regression model

Note: *p < 0.05, **p < 0.01, ***p < 0.001

## Discussion

In this research, we found a strong correlation between nurses’ willingness to remain in their occupations and three dimensions of Maslow’s hierarchy of needs: “self-actualization needs”, “beyond self-actualization needs” and “medical humanities education-relevant needs”. Additionally, regarding retention, nursing staff with less than 1 year or 1–3 years of work experience had substantially greater risk of turnover than senior staff with 5–10 years of work experience.

In the survey, the participants under the age of 29 years represented the majority (57.9%) of the participants. Regarding retention, age may be a high-risk factor since it involves a reliance on recruitment budgets and political development [19]. Because not all nurse managers arrange training after recruitment, a lack of training may negatively influence the socialization process of newly hired nurses and contribute to inadequate retention [20]. The nurses’ intention to remain in their present occupations was related to multiple demographic and participant characteristics in the results of this research. Notably, younger nurses had lower intention to stay. Because the association between age and intention to leave was not strong, this finding can be explained by the correspondence of age and years of experience. Nurses with more experience are commonly older. The direct connection between years of experience/age and intention to stay is discussed in other studies in the literature [21, 22].

Our findings suggest that nurse planners, policy makers, and decision makers should implement targeted retention programs designed to retain newly graduated nurses and inexperienced nurses [23]. The present study findings further indicated that nurses with less than 1 year of work experience were approximately four times as likely to indicate intention to stay in their current jobs than their counterparts with 5 ~ 10 years of experience in nursing (Table 4). Such findings are also in accordance with previously reported studies in the literature [24]. Sociocultural factors associated with the role of experienced employees in society may also play a role in enhancing nurse retention [25]. Older nurses generally also display higher levels of job commitment [26].

**Table 4:** Logistic regression of intention to stay (binary)

Seniority of nursing work	OR	95% CI	*P*-value
Within1 year	3.316	(1.491, 8.158)	0.005**
1 ~ 3 years	2.348	(1.221, 4.622)	0.011*
3 ~ 5 years	1.287	(0.676, 2.479)	0.444
Over 10 years	2.750	(1.434, 5.399)	0.003**
Model adjusted age and sex			
Within1 year	4.511	(1.818, 12.069)	0.002**
1 ~ 3 years	3.248	(1.500, 7.102)	0.003**
3 ~ 5 years	1.698	(0.820, 3.554)	0.156
Over 10 years	1.566	(0.730, 3.406)	0.250

Note: reference is 5 ~ 10 year of seniority of nursing work

Note: intention to stay as response scores ≥3

Note: *p < 0.05, **p < 0.01, ***p < 0.001

The present study, which drew on Maslow’s hierarchy of needs, revealed that influencing factors of nurse retention in Taiwan are related to nurses’ professional qualifications. This information allows administrators and medical practitioners to make informed decisions regarding nursing retention. Many medical institutions seem to follow a standard strategy during the recruitment period [27], but not all institutions conduct job analysis before starting recruitment. This may be because most interviewing managers are not involved in regular budgeting plans, which can support effective analysis of the main weaknesses of nursing staff.

Within Maslow’s hierarchy of needs, physiological needs, safety needs, love and belonging needs (social needs) are also collectively called “deficiency needs.” For nursing staff, basic needs such as physiological and safety needs are not met, which may ultimately lead to the loss of nursing staff. The results of this study showed that the participants’ satisfaction of their physiological needs was significantly lower than their satisfaction of other needs. This finding demonstrated that the satisfaction of hospital nursing staff with their “working time” and “salary structure” was lower than their satisfaction with other areas. Even with the adjustment of salary structure in hospitals and improvements in the work process, salary and working hours are still important issues for nurses in the workplace.

Salary may not be the primary reason for leaving. A sense of achievement from work, positive relationships with colleagues, and an ethical culture are important factors contributing to retention [28, 29]. The satisfaction of love and belonging needs (social needs) was reported to be highest across all dimensions. Clearly, hospital employees work through “unit cooperation” and “cooperation with other teams.”

With regard to Maslow’s high-level needs, esteem needs and self-fulfillment needs are known as advanced “growth needs,” and Maslow later added a spiritual need, that is, “beyond self-fulfillment needs.” If people’s needs are not being satisfied, this will determine their intention to stay. Although there was no significant difference between the groups included in the analysis of medical and human-related education and training hours in the past 6 months, the regression coefficient in the regression model was significant. The promotion of human activities in hospitals should not take a long time.

According to Maslow’s theory, people are motivated by a hierarchy of needs. Therefore, when a job meets all levels of people’s needs, employee satisfaction will increase accordingly, and willingness to stay at one’s current job will follow. Feedback from nursing staff regarding clinical work indicates that the work meets their basic needs (i.e., physiological needs, safety needs, and love and belonging needs), further satisfying self-esteem and self-actualization needs, and even high-level needs for spiritual satisfaction. After having their various needs met, nursing staff will be less likely to resign from their current positions. Health coverage organizations should be able to provide a variety of resources to meet the needs of their nursing staff at all levels in the medical workplace and to attract new nursing staff to maintain clinical operations in hospitals and clinics. Based on the theory of Maslow’s hierarchy of needs, this study explored the needs of nursing staff and the relationship between each level of needs and turnover rate. Moreover, a hospital’s education and training in medicine and the humanities also affects the clinical practice of nursing staff and their intention to stay.

### Limitations

The data used in this study were limited to the available data from a faith-based hospital. The present study was conducted at one specific time, not as longitudinal research. In addition, the nurse employees responded to a self-report questionnaire. Data on the professional characteristics of the nurses in the study (shift work, type of contract, nurse rate, and so forth) were not collected in this study. The potential biases of the method was minimized by the substantial sample size and high response rate (96.04%).

## Conclusion

We believe that to increase the retention of nursing staff, several strategies could be pursued, including providing a regular day shift system, providing strong basic education and training, cultivating a working environment that values lifelong learning, tailoring a promotion path, strengthening the communication skills of clinical instructors and head nurses, and funding further research investigating ways to improve the retention of nursing staff. Regarding ​​medical humanities education, to make nursing staff more willing to accept the curriculum, we recommend requiring training courses that can be completed during public leave in the curriculum. In addition, it is advisable to tailor different educational programs to different groups (e.g., nurses with 3–5 years of work experience and those with 5–10 years of work experience in the case study hospital) to enhance the effectiveness of medical humanities education.

## Supplementary Information

**Additional file 1.** English version of the developed questionnaire.

## Data Availability

Datasets used in the analysis are available from the corresponding author on reasonable request.
