# Supplementary material for: Exploring factors influencing the retention of nurses in a religious hospital in Taiwan: a cross-sectional quantitative study
Source: BMC Nurs. 2021 Mar 12;20:42. doi: 10.1186/s12912-021-00558-7 (PMC7953797; doi:10.1186/s12912-021-00558-7)
Supplement: Supplementary file 1 — Additional file 1. English version of the developed questionnaire. [file 12912_2021_558_MOESM1_ESM.pdf]

Questionnaire

Dear nursing colleagues,

Thank you for taking the time to fill out this questionnaire. This research aims to understand the main factors that affect the retention of nurses in a religious hospital. Your opinion will be a valuable reference to the study. This survey is anonymous, and all data obtained are for academic research purposes only. So please feel free to answer the questions. Your assistance will enable the successful completion of this research. Your participation is very much appreciated.

Graduate Institute of Public Affairs,
College of Social Sciences, National Taiwan University, Taipei, Taiwan
No. 1, Sec. 4, Roosevelt Road, Taipei, 10617 Taiwan R.O.C.
Supervisor：Tsai-Tsu Su, PhD
Researcher：Li-Hua Chiao

Part I：Survey Questions

★Instructions: Please circle or tick a score that you think is the most appropriate.
(5：Strongly Agree; 4：Agree; 3：Neutral; 2：Disagree; 1：Strongly Disagree)

**Question number**	**Item**	Strongly Agree	Agree	Neutral	Disagree	Strongly Disagree
**1**	I think my weekly total working hours is reasonable.	5	4	3	2	1
**2**	I think my current salary is reasonable.	5	4	3	2	1
**3**	My working environment can give me a sense of security.	5	4	3	2	1
**4**	My direct supervisor	5	4	3	2	1

Question number	Item	Strongly Agree	Agree	Neutral	Disagree	Strongly Disagree
	will guide and assist me in time to deal with work problems.					
**5**	The colleagues in the unit help each other in the department I serve.	5	4	3	2	1
**6**	I work smoothly with other team members at work.	5	4	3	2	1
**7**	I get trust from my supervisor at work.	5	4	3	2	1
**8**	My immediate supervisor is willing to accept my constructive suggestions at work.	5	4	3	2	1
**9**	I am satisfied with the resources and opportunities for learning and growth provided by the hospital for staff.	5	4	3	2	1
**10**	My current job can realize or inspire my ideals in life.	5	4	3	2	1
**11**	I always feel joy and satisfaction from the bottom of my heart.	5	4	3	2	1
**12**	Religion brings me spiritual satisfaction.	5	4	3	2	1
**13**	I am satisfied with the medical humanities education (i.e., holistic medical care, humanistic care, spiritual care) and	5	4	3	2	1

Question number	Item	Strongly Agree	Agree	Neutral	Disagree	Strongly Disagree
	training provided by the hospital.					
**14**	Medical humanities education (i.e., holistic medical care, humanistic care, spiritual care) and training can improve my nursing service quality.	5	4	3	2	1
**15**	I am willing to continue to serve and make contributions to the current hospital.	5	4	3	2	1
**16**	I am willing to make extra efforts to grow together with the current hospital.	5	4	3	2	1

Part II：General Information

1.Sex： □Female □Male

2.Age bands： □Below 29 □30~39 □40~49 □Over 50 Y/O

3.Education level：□Junior high school □Junior college □Two-year college □Two-year technical college □University □Graduate school

4.Religion：□None □Buddhism □Taoism □Christianity □Catholic □Others

5.Seniority of nursing work：□Within1 □1~3 □ 3~5 □5~10 □Over 10 Years

6.Seniority of hospital work：□Within1 □1~3 □3~5 □5~10 □Over 10 Years

7.Time of received medical and human-related education and training in the past six months： □None □1-5 □6-10 □Over 10 Hours
